# Supplementary material for: In Vivo Absorption of Iron Complexes of Chondroitin Sulfates with Different Molecular Weights and Their Anti-Inflammation and Metabolism Regulation Effects on LPS-Induced Macrophages
Source: Foods. 2025 Sep 27;14(19):3356. doi: 10.3390/foods14193356 (PMC12524291; doi:10.3390/foods14193356)
Supplement: Supplementary file 1 [file foods-14-03356-s001.zip › foods-3844408-supplementary.pdf]

Supplementary Materials

In Vivo Absorption of Iron Complexes of Chondroitin Sulfates with Different Molecular Weights and Their An-ti-Inflammation and Metabolism Regulation Effects on LPS-Induced Macrophages

Qianqian Du[1], Jiachen Zheng[1], Fanhua Kong[1], Xiuli Wu[2], Chunqing, Ai[1], Shuang Song[1 *]

[1] SKL of Marine Food Processing & Safety Control, National Engineering Research Center of Seafood, Col-laborative Innovation Center of Seafood Deep Processing, National & Local Joint Engineering Laboratory for Marine Bioactive Polysaccharide Development and Application, Liaoning Key Laboratory of Food Nutrition and Health, School of Food Science and Technology, Dalian Polytechnic University, Dalian 116034, China; songs1008@163.com

[2] College of Pharmacy, Ningxia Medical University, Yinchuan 750000, P. R. China.

[*] Correspondence: songs1008@163.com

Table S1. Mass spectrometric parameters for targeted metabolite analysis method

Q1 (Da)	Q3 (Da)	Time (msec)	ID	CE (volts)
61.04	61.04	3	Urea	21
62.06	62.061	3	Ethanolamine	53
76.04	58.066	3	Glycine	20
89.1	72.081	3	Putrescine	20
90.06	90.055	3	Alanine	20
90.06	72.045	3	Sarcosine	20
103.1	95.049	3	Betaine aldehyde	20
103.039	95.049	3	Acetoacetic acid	20
104.107	60.081	3	Dimethylglycine	21
104.07	58.066	3	Choline	20
106.05	60.045	3	Serine	20
112.05	70.066	3	Cytosine	20
113.035	70.066	3	Uracil	22
114.07	86.072	3	Creatinine	20
116.07	70.066	3	Proline	20
118.07	91.055	3	Indole	20
118.09	72.081	3	Valine	20
118.09	58.066	3	Betaine	21
120.066	74.061	3	Threonine	20

120.07	103.054	3	Homoserine	32
121.05	94.074	3	Purine	35
122.03	76.065	3	Cysteine	20
123.06	80.05	3	Nicotinamide	22
127.05	81.045	3	Imidazoleacetic acid	20
127.05	85.076	3	Thymine	20
130.09	84.081	3	DL-Pipecolic acid	20
131.11	114.091	3	N-Acetyl-putrescine	20
132.066	68.05	3	Hydroxyproline	20
132.08	90.055	3	Creatine	20
132.1	86.1	3	Leucine	20
132.1	86.097	3	Isoleucine	20
133.06	74.024	3	Asparagine	20
133.097	70.066	3	Ornithine	20
134.045	58.066	3	Aspartate	20
136.04	119.049	3	Methylcysteine	20
137.07	94.073	3	2-methylnicotinamide	27
140.995	123.055	3	Acetylphosphate	24
142.1	124.058	3	Histidinol	20
146.17	87.044	3	Spermidine	20
147.08	130.05	3	Glutamine	20
147.11	130.05	3	Lysine	32

148.06	84.045	3	Glutamate	20
148.06	106.05	3	O-acetyl-L-serine	20
150.06	104.053	3	L-methionine	20
152.06	135.027	3	Guanine	20
156.08	110.071	3	Histidine	20
160.13	114.128	3	2-Aminooctanoicacid	20
162.11	103.039	3	L-Carnitine	20
166.09	120.081	3	Phenylalanine	30
169.1	151.048	3	Pyridoxamine	20
170.08	152.07	3	Pyridoxine	24
170.09	96.069	3	1-Methyl-Histidine	20
175.11	116.071	3	N-acetylornithine	20
175.12	116.071	3	Arginine	20
176.1	159.076	3	Citrulline	20
182.08	136.075	3	Tyrosine	39
184.072	126.091	3	Phosphorylcholine	20
189.09	148.06	3	N-Acetylglutamine	20
189.12	126.091	3	N6-Acetyl-L-lysine	20
190.07	130.05	3	N-acetyl-glutamate	20
203.15	116.071	3	Ng,NG-dimethyl-L-arginine	24
203.15	144.102	3	L-Acetylcarnitine	27
205.1	146.06	3	Tryptophan	20

209.09	94.066	3	Kynurenine	25
215.03	72.081	3	Deoxyribose1-phosphate	25
221.1	126.055	3	N-Acetylgalactosamine	23
223.09	134.027	3	Cystathionine	20
223.08	165.054	3	Flavone	29
231.03	116.071	3	Ribose-5-phosphate	20
231.03	100.076	3	D-Ribulose5-phosphate	20
235.11	118.086	3	5-Methoxytryptophan	22
244.09	162.049	3	Cytidine	20
245.1	227.084	3	Biotin	20
260.05	144.103	3	Glucosamine6-phosphate	20
261.04	98.985	3	Glucose6-phosphate	20
265.11	122.071	3	Thiamine	20
282.12	150.077	3	1-Methyladenosine	27
284.1	152.057	3	Guanosine	35
291.13	84.081	3	Argininosuccinic acid	37
298.1	136.062	3	5-Methylthioadenosine	29
298.11	177.033	3	7-methylguanosine	24
308.06	76.022	3	dCMP	20
324.05	112.05	3	Cytidine monophosphate	20
348.07	136.061	3	3-AMP	23
364.07	152.056	3	Guanosine monophosphate	21

377.15	91.055	3	Riboflavin	26
399.15	250.093	3	S-Adenosylmethionine	20
442.15	295.093	3	Folate	30
444.16	89.06	3	Dihydrofolic acid	32
488.107	360.209	3	CDP-choline	21
492.009	136.062	3	dATP	29
500.305	59.05	3	Taurodeoxycholic acid	32
508.99	136.062	3	Inosine triphosphate	25
523.998	184.073	3	Guanosine triphosphate	25
663.12	136.062	3	NAD	41
664.12	136.062	3	NADH	53
688.157	184.073	3	Dephospho-CoA	37
743.08	136.062	3	NADP+	40
744.083	136.062	3	NADPH	56
768.123	184.073	3	Coenzyme-A	20
786.16	439.101	3	FAD	26
808.12	184.073	3	Acetyl-CoA	53
824.149	184.073	3	Propionyl-CoA	33
838.165	184.073	3	Butyryl-CoA	28
852.144	184.073	3	Acetoacetyl-CoA	32
854.123	184.073	3	Malonyl-CoA	26
854.16	184.073	3	3-Hydroxybutyryl-CoA	33

868.139	184.073	3	Succinyl-CoA	22
69	42.24	3	Imidazole	23
102	58	3	Betaine aldehyde	21
104.01	69	3	4-Aminobutyrate	22
127.002	81	3	Imidazoleacetic acid	15
130	84	3	DL-Pipecolic acid	18
132.1	86	3	leucine-isoleucine	13
136	119	3	Adenine	26
136.12	90.1	3	Homocysteine	17
137.001	94	3	Methyl-nicotinamide	20
150.1	133	3	Methionine	12
160	55.3	3	2-Aminooctanoic acid	21
162.1	103	3	Carnitine	20
166	74	3	Methionine sulfoxide	14
175	115.1	3	N-acetyl-L-ornithine	16
177.05	74	3	N-carbamoyl-L-aspartate	19
180	162	3	Glucosamine	12
186	88	3	3-Phospho-serine	12
189.002	84	3	Acetyl-lysine	23
189.1	130	3	N-acetyl-glutamine	17
190.1	84.1	3	N-acetyl-glutamate	24
204	85	3	Acetyl-carnitine DL	19

222	138	3	N-acetyl-glucosamine	18
241.002	74	3	Cystine	32
252	136	3	Deoxyadenosine	22
258.1	104	3	Glycerophosphocholine	16
259	110	3	Acadesine	24
260	126	3	D-glucosamine-6-phosphate	17
260.1	162.1	3	D-glucosamine-1-phosphate	17
268	88	3	S-ribosyl-L-homocysteine_pos	31
268.1	152	3	deoxyguanosine	17
268.15	136.1	3	adenosine	29
291	70	3	L-arginino-succinate	37
298	136	3	S-methyl-5-thioadenosine	29
308.1	162	3	Glutathione	21
323	81	3	dTMP	19
324	112	3	CMP	18
325	97	3	UMP	14
332.1	136	3	dAMP	23
335	123	3	Nicotinamide ribotide	30
339	110	3	Aminoimidazole carboxamide ribonucleotide	32
345.2	122	3	Thiamine-phosphate	15
348.1	135	3	dGMP	38
348.15	136	3	AMP	23

349	137	3	IMP	21
355	250	3	S-adenosyl-L-methioninamine	20
364	152	3	GMP	21
365	97	3	xanthosine-5-phosphate	13
385.1	136	3	S-adenosyl-L-homo-cysteine	21
399.1	250	3	S-adenosyl-L-methionine	15
444.2	178	3	7,8-Dihydrofolate	32
460.1	313.1	3	5-Methyl-THF	21
525.5	352.8	3	Diiodothyronine	31
613	231	3	Glutathione disulfide	35
664.1	428	3	NAD^+	32
688	348	3	Dephospho-CoA	27
744.2	136	3	$NADP^+$	50
768	261	3	Coenzyme A	39
810	303	5	Acetyl-CoA	30
824.1	317.1	3	Propionyl-CoA	35
852	345	3	Acetoacetyl-CoA	36
854	347	3	Malonyl-CoA	28
868.1	361.1	3	Succinyl-CoA	40
202.1	129.1	3	Spermine	19
171	108	3	L-phenylalanine-d5	30
151.15	107	3	DL-glutamic-2,4,4-D3 acid	17

Table S2. Chromatographic peak area data of LPS-induced macrophage metabolites by CS, CS-Fe, and LCS-Fe

No	Metabolite name	Normalized peak area value					
		Blank-1	Blank-2	Blank-3	Blank-4	Blank-5	Blank-6
1	Glycine	0.003250568	0.003426242	0.003886713	0.003223593	0.003531349	0.004082351
2	Alanine	0.00109872	0.001938881	0.001414983	0.002065824	0.001429203	0.001336635
3	Proline	0.019902349	0.020599636	0.018211055	0.019443716	0.02055398	0.018698401
4	Valine	0.000383311	0.000169437	0.000262322	0.000652664	0.000363314	0.000229148
5	Betaine	0.593121766	0.594866246	0.601816648	0.604991269	0.588872267	0.605289193
6	Threonine	0.000536537	0.000392932	0.000384067	0.000466626	0.000489722	0.000424898
7	Homoserine	0.001226545	0.001709215	0.002448708	0.00188251	0.00200488	0.002424779
8	Pipecolic acid	0.00392185	0.00392932	0.004890048	0.005646964	0.004795426	0.005270471
9	Creatine	0.029257146	0.041558614	0.037476555	0.037286985	0.033179672	0.033180425
10	Leucine	0.000457156	0.000539012	0.00073627	0.000511008	0.000619032	0.000867907
11	Isoleucine	0.000694079	0.001043652	0.000573268	0.001243467	0.000650323	0.000804879
12	Methylcysteine	0.001102791	0.001291285	0.000970182	0.001290572	0.001265585	0.000839653
13	2-Methylnicotinamide	0.00048728	0.001016702	0.001245074	0.00103575	0.001103099	0.001293892
14	Spermidine	0.007547342	0.007022781	0.006584528	0.004843903	0.00680106	0.00703454
15	Glutamate	0.001494813	0.000926866	0.001042013	0.001534612	0.001012619	0.001576795
16	Guanine	0.000840629	0.001097554	0.001070101	0.001061856	0.000861819	0.00152246
17	2-Aminooctanoic acid	0.001164261	0.001131925	0.000712787	0.001387621	0.001199987	0.001398577
18	Phenylalanine	0.000873603	0.000728448	0.000657071	0.000771846	0.001008095	0.00099976

19	Citrulline	0.000125952	6.88E-05	0.000150753	0.000158172	8.78E-05	1.70E-05
20	5-Methoxytryptophan	0.00908205	0.009948288	0.007256794	0.005452867	0.01031092	0.009407158
21	Deoxycytidine monophosphate	0.001102791	0.001260038	0.001430638	0.001388756	0.00179263	0.001584039
22	Guanosine monophosphate	0.003881548	0.00254898	0.003129262	0.003027226	0.003443885	0.003292324
23	Guanosine triphosphate	0.000498271	0.000506593	0.000390191	0.000587965	0.000424878	0.0004057
24	NADH	0.002431921	0.002593117	0.003220893	0.002011341	0.002811281	0.003469093
25	Dephospho-CoA	0.000652556	0.000559323	0.000727521	0.000712255	0.000618655	0.000721566
26	NADPH	0.001032773	0.001001469	0.001213303	0.000919973	0.001058613	0.001313814
27	Acetyl-CoA	0.023940625	0.020377001	0.027862223	0.029091794	0.024866847	0.016007019
28	Butyryl-CoA	0.001048242	0.000540574	0.001199489	0.00099432	0.001088019	0.000450978
29	3-Hydroxybutyryl-CoA	0.000208386	0.000147096	0.000129941	0.000290691	0.000132138	0.000136416
30	Betaine aldehyde	0.001485043	0.001576415	0.001366635	0.001243467	0.001123834	0.001571723
31	Homocysteine	0.000306616	0.000241149	0.000410543	0.000274913	0.000292551	0.000492273
32	Carnitine	0.018672955	0.017838176	0.016682339	0.014074844	0.02039941	0.017111826
33	Glucosamine	0.000584166	0.000594085	0.000732126	0.000839383	0.00083166	0.0007759
34	Acetylcarnitine	0.00604113	0.009143676	0.005741893	0.005771821	0.006424061	0.005741373
35	Glycerophosphocholine	0.013999628	0.011420807	0.011746245	0.012241709	0.012637003	0.014442178
36	S-Methyl-5-thioadenosine	0.013698385	0.012373843	0.009407126	0.010164534	0.012395724	0.010425029
37	Glutathione	0.006155114	0.00655798	0.006800943	0.006407459	0.007019719	0.005951467
38	Uridine monophosphate	0.001397927	0.000938584	0.001203173	0.000914297	0.00118717	0.001080899
39	Adenosine monophosphate	0.047140357	0.044839557	0.048900481	0.042644507	0.043581072	0.04100463
40	NAD^+	0.001138208	0.001518999	0.001473921	0.000694662	0.001101591	0.001295703

41	$NADP^+$	0.000860169	0.000728838	0.00068608	0.000564526	0.001026945	0.000908839
42	Hydroxyproline	0.000457156	0.000539012	0.00073627	0.000511008	0.000619032	0.000867907
		**Model-1**	**Model-2**	**Model-3**	**Model-4**	**Model-5**	**Model-6**
1	Glycine	0.004868219	0.005616216	0.005128909	0.004837867	0.004716989	0.004372575
2	Alanine	0.002592799	0.002238172	0.002294309	0.002572408	0.002156635	0.002273902
3	Proline	0.035797908	0.032971648	0.033868554	0.03560386	0.034583163	0.035730417
4	Valine	0.001319202	0.001188627	0.001331047	0.001150324	0.001203873	0.000871255
5	Betaine	0.366358494	0.370194053	0.385459338	0.369937118	0.388111119	0.401559685
6	Threonine	0.000541696	0.000649682	0.000618634	0.000735439	0.000469618	0.000760412
7	Homoserine	0.004957204	0.005302524	0.004210414	0.005106966	0.00422448	0.003363174
8	Pipecolic acid	0.013329205	0.011663286	0.011550659	0.012130152	0.011573966	0.012559437
9	Creatine	0.056282987	0.051967006	0.0562289	0.063075609	0.056152985	0.057092056
10	Leucine	0.002360326	0.002751039	0.002608064	0.002405331	0.002038364	0.002297945
11	Isoleucine	0.002166413	0.002782786	0.002471061	0.002108139	0.002406358	0.002018394
12	Methylcysteine	0.001097852	0.001302766	0.001896037	0.001648896	0.001408854	0.00132196
13	2-Methylnicotinamide	0.000587672	0.000300502	0.0003498	0.000379696	0.000854954	0.000394469
14	Spermidine	0.00331951	0.003690224	0.003863084	0.003817656	0.003690351	0.00348828
15	Glutamate	0.005116635	0.00435389	0.004368642	0.003793999	0.004370152	0.004441852
16	Guanine	0.000460868	0.001058615	0.000596636	0.000817351	0.001047796	0.000949497
17	2-Aminooctanoic acid	0.00168886	0.002382923	0.002291222	0.002034211	0.001703319	0.001925482
18	Phenylalanine	0.00127471	0.001469816	0.001329117	0.001726965	0.001127222	0.001054227
19	Citrulline	0.000475328	0.00045542	0.000699291	0.000590539	0.000709283	0.000564808

20	5-Methoxytryptophan	0.002576485	0.002643703	0.002685634	0.004089713	0.003051823	0.002236004
21	Deoxycytidine monophosphate	0.002417425	0.002494794	0.002525091	0.00281164	0.002019635	0.001822382
22	Guanosine monophosphate	0.002560913	0.002683387	0.002226773	0.002492862	0.002195134	0.002524928
23	Guanosine triphosphate	0.000792337	0.000960728	0.000908076	0.000691082	0.000849058	0.000575403
24	NADH	0.00110675	0.001356055	0.000997995	0.001425633	0.001510824	0.001362304
25	Dephospho-CoA	0.00098625	0.000996255	0.001151593	0.000932679	0.00100895	0.00076734
26	NADPH	0.001102672	0.001130424	0.001214112	0.001596851	0.001048143	0.001098645
27	Acetyl-CoA	0.022794981	0.022593364	0.022009157	0.014002016	0.017581191	0.020045383
28	Butyryl-CoA	0.001961377	0.001984195	0.001490818	0.000807297	0.000910101	0.001580729
29	3-Hydroxybutyryl-CoA	0.000606952	0.000423295	0.000263971	0.000292312	0.000358977	0.000184642
30	Betaine aldehyde	0.000603244	0.000374654	0.000728622	0.000603255	0.000656216	0.000695618
31	Homocysteine	0.000915062	0.000564267	0.000541063	0.000388863	0.000648586	0.00039288
32	Carnitine	0.007166997	0.00721869	0.007675997	0.008025654	0.007640829	0.007115113
33	Glucosamine	0.000946578	0.000798969	0.001047008	0.001091477	0.000875765	0.00086555
34	Acetylcarnitine	0.00510922	0.005960143	0.005256264	0.004592425	0.004807167	0.004678207
35	Glycerophosphocholine	0.036639557	0.040288602	0.036446516	0.040128271	0.039504787	0.037780189
36	S-Methyl-5-thioadenosine	0.105632563	0.105370191	0.093856339	0.090931786	0.094755994	0.102244089
37	Glutathione	0.008601879	0.007169557	0.008521168	0.009643205	0.00879233	0.00788938
38	Uridine monophosphate	0.000622524	0.000686342	0.000851731	0.001029969	0.000872643	0.000841099
39	Adenosine monophosphate	0.041415083	0.04712935	0.042760207	0.047491528	0.043840255	0.040274146
40	NAD^+	0.000575807	0.000479608	0.000452687	0.000473141	0.000486613	0.000538727
41	$NADP^+$	0.000846099	0.00078234	0.001058199	0.000956041	0.001083173	0.000494716

42	Hydroxyproline	0.002360326	0.002751039	0.002608064	0.002405331	0.002038364	0.002297945
		**CS-1**	**CS-2**	**CS-3**	**CS-4**	**CS-5**	**CS-6**
1	Glycine	0.006174167	0.005385935	0.006532606	0.005064337	0.004934352	0.005975049
2	Alanine	0.002399616	0.001855372	0.002580357	0.002087328	0.002038528	0.002281152
3	Proline	0.033418133	0.031225133	0.030572059	0.032293701	0.031692851	0.031054343
4	Valine	0.001317924	0.001179199	0.001221002	0.001231866	0.001096857	0.001231902
5	Betaine	0.409993793	0.407267354	0.418194244	0.433719371	0.429778262	0.406896524
6	Threonine	0.00061681	0.000297633	0.000186262	0.000254126	0.000496449	0.000594973
7	Homoserine	0.006965649	0.007113499	0.006702749	0.00722331	0.006633125	0.006003984
8	Pipecolic acid	0.012536349	0.010912576	0.013132374	0.013152732	0.012042078	0.011834359
9	Creatine	0.052280274	0.052921305	0.050908657	0.051327736	0.051867193	0.054108195
10	Leucine	0.003266001	0.003049815	0.003422566	0.003182854	0.003333889	0.003319552
11	Isoleucine	0.003156831	0.003408226	0.003298988	0.003633041	0.003327109	0.003425888
12	Methylcysteine	0.002548208	0.001039274	0.002175595	0.001250044	0.000979714	0.002402317
13	2-Methylnicotinamide	0.001785838	0.000959931	0.002216788	0.001014257	0.001594813	0.001275304
14	Spermidine	0.004251563	0.003401581	0.003776285	0.003704152	0.003695114	0.003159687
15	Glutamate	0.004366798	0.003489914	0.004327907	0.002865799	0.00356893	0.003927908
16	Guanine	0.001001937	0.001049827	0.001138169	0.000576902	0.001003444	0.000599313
17	2-Aminooctanoic acid	0.001647253	0.002355272	0.001550095	0.002885581	0.00176243	0.002523482
18	Phenylalanine	0.00154809	0.00144107	0.001497261	0.001153805	0.001033954	0.001352343
19	Citrulline	0.000754789	0.000515142	0.000373912	0.000445375	0.000762376	0.000459703
20	5-Methoxytryptophan	0.00446687	0.002732443	0.002884824	0.003058278	0.004278949	0.003938758

21	Deoxycytidine monophosphate	0.002801422	0.002075813	0.003085862	0.002193191	0.002499193	0.002315151
22	Guanosine monophosphate	0.002979126	0.002145384	0.001853666	0.001533416	0.002059998	0.001826513
23	Guanosine triphosphate	0.00072689	0.000827042	0.001011905	0.000626091	0.000623762	0.000885769
24	NADH	0.001770373	0.001901102	0.001673225	0.001484227	0.001777873	0.001658329
25	Dephospho-CoA	0.001011338	0.001434425	0.001069216	0.000931385	0.00101851	0.0010384
26	NADPH	0.001339151	0.000823915	0.000809524	0.001062377	0.001065217	0.001187414
27	Acetyl-CoA	0.024535945	0.031928666	0.031821269	0.029310276	0.027541969	0.023730205
28	Butyryl-CoA	0.001319137	0.001883905	0.002057838	0.002675458	0.001875054	0.001473508
29	3-Hydroxybutyryl-CoA	0.000213397	0.000515142	0.000252887	0.000502851	0.000335574	0.000357526
30	Betaine aldehyde	0.000571626	0.000542893	0.000565502	0.000845838	0.000429402	0.000519381
31	Homocysteine	0.001187223	0.001228837	0.001504872	0.001369809	0.001320222	0.001242029
32	Carnitine	0.005507017	0.006112919	0.005981879	0.005485652	0.005359987	0.005211892
33	Glucosamine	0.001201476	0.000941171	0.001345923	0.000820174	0.000919823	0.00084743
34	Acetylcarnitine	0.005212865	0.0050029	0.004898335	0.005240241	0.00551442	0.005031049
35	Glycerophosphocholine	0.065259367	0.060269315	0.058027809	0.066886456	0.065276576	0.054397544
36	S-Methyl-5-thioadenosine	0.068898365	0.073519184	0.059281497	0.065068599	0.066707914	0.074941297
37	Glutathione	0.010083057	0.010232495	0.010741413	0.008094812	0.008309298	0.008362176
38	Uridine monophosphate	0.000999208	0.000817271	0.001135035	0.000659241	0.000963894	0.000483574
39	Adenosine monophosphate	0.042060754	0.040257713	0.041676146	0.035592577	0.038307142	0.039206741
40	NAD^+	0.000716579	0.000717604	0.001105931	0.000607378	0.000761623	0.00059642
41	$NADP^+$	0.000700204	0.000600739	0.000730273	0.000623418	0.000693069	0.000807644
42	Hydroxyproline	0.003266001	0.003049815	0.003422566	0.003182854	0.003333889	0.003319552

		CS-Fe-1	CS-Fe-2	CS-Fe-3	CS-Fe-4	CS-Fe-5	CS-Fe-6
1	Glycine	0.004683789	0.006163193	0.006795392	0.004959346	0.005493967	0.004486549
2	Alanine	0.003216317	0.003845075	0.004454805	0.003413644	0.00393138	0.004053149
3	Proline	0.023622401	0.024181669	0.027249907	0.023363505	0.024884687	0.026069829
4	Valine	0.000416823	0.000377902	0.000696197	0.000823868	0.000545138	0.000719096
5	Betaine	0.455392448	0.436231667	0.44889737	0.437514015	0.434832549	0.447961427
6	Threonine	0.000252284	0.000708597	0.000500578	0.000438653	0.000598374	0.000406008
7	Homoserine	0.003735776	0.003734341	0.003820112	0.004126936	0.004748661	0.004209173
8	Pipecolic acid	0.01194323	0.010247705	0.012505913	0.010787172	0.010902755	0.014461665
9	Creatine	0.097311998	0.096454697	0.094007908	0.091783502	0.101617087	0.098364285
10	Leucine	0.001745382	0.001471104	0.001590147	0.001646788	0.002232935	0.001912157
11	Isoleucine	0.001825899	0.002335602	0.002037336	0.00212801	0.001691631	0.001703432
12	Methylcysteine	0.002156621	0.000821759	0.002489651	0.001037903	0.001689927	0.000708694
13	2-Methylnicotinamide	0.00167742	0.001732396	0.001757576	0.001591737	0.001522979	0.001682282
14	Spermidine	0.004086411	0.003592525	0.003877773	0.003255609	0.003502936	0.004462278
15	Glutamate	0.004242682	0.003738227	0.003879908	0.004198597	0.003554895	0.00441027
16	Guanine	0.001017707	0.001262265	0.001162606	0.00107587	0.000817707	0.00136122
17	2-Aminooctanoic acid	0.000931563	0.000889268	0.000624014	0.000981429	0.000858166	0.000712162
18	Phenylalanine	0.001157095	0.00120447	0.0009089	0.001010378	0.001301516	0.000910138
19	Citrulline	0.000519892	0.000438951	0.000723959	0.000469643	0.000520436	0.000613
20	5-Methoxytryptophan	0.003169566	0.00323944	0.003299033	0.003388491	0.003056179	0.004306255
21	Deoxycytidine monophosphate	0.002351418	0.002606122	0.002533216	0.002264689	0.003030199	0.002628133

22	Guanosine monophosphate	0.002655302	0.002350173	0.00308035	0.003157371	0.003067252	0.003689094
23	Guanosine triphosphate	0.001098656	0.000867412	0.000562937	0.000802987	0.000643092	0.000760702
24	NADH	0.001636296	0.001532785	0.001812247	0.001353024	0.001601768	0.001859456
25	Dephospho-CoA	0.001163155	0.000823216	0.001034899	0.000849021	0.000880738	0.000800575
26	NADPH	0.001419422	0.001174358	0.001329608	0.001172684	0.001243596	0.001139667
27	Acetyl-CoA	0.025622318	0.026187499	0.021718774	0.022328923	0.024113829	0.014135749
28	Butyryl-CoA	0.001767892	0.001688686	0.001261269	0.001521499	0.001280648	0.000540189
29	3-Hydroxybutyryl-CoA	0.000486127	0.000318262	0.000219921	0.000134021	7.32E-05	0.000195862
30	Betaine aldehyde	0.000296914	0.000464255	0.000541581	0.000494985	0.000409109	0.000363362
31	Homocysteine	0.000569673	0.000440603	0.000848677	0.000545291	0.000856888	0.000597744
32	Carnitine	0.004796339	0.005065571	0.005471337	0.004693108	0.004940311	0.004937284
33	Glucosamine	0.00100169	0.001151045	0.001626452	0.001068276	0.000987636	0.000807163
34	Acetylcarnitine	0.004493321	0.004401656	0.004911817	0.005899012	0.005804865	0.004743121
35	Glycerophosphocholine	0.048569423	0.047401704	0.052705933	0.05234602	0.049743821	0.050378325
36	S-Methyl-5-thioadenosine	0.047443928	0.052452706	0.04557312	0.053105346	0.04901981	0.050517012
37	Glutathione	0.011155383	0.010111716	0.012356423	0.007711902	0.009569723	0.010394647
38	Uridine monophosphate	0.001221595	0.000881983	0.000967414	0.001031259	0.001026392	0.000793294
39	Adenosine monophosphate	0.044327174	0.043822293	0.039623415	0.042901906	0.039969672	0.039699368
40	NAD^+	0.000732437	0.000683342	0.000427542	0.000603189	0.000865406	0.000586996
41	$NADP^+$	0.000779189	0.000858185	0.000939225	0.001086785	0.000679719	0.000693439
42	Hydroxyproline	0.001745382	0.001471104	0.001590147	0.001646788	0.002232935	0.001912157
		**LCS-Fe-1**	**LCS-Fe-2**	**LCS-Fe-3**	**LCS-Fe-4**	**LCS-Fe-5**	**LCS-Fe-6**

1	Glycine	0.005388721	0.006348934	0.004899239	0.005392401	0.005085513	0.005300457
2	Alanine	0.003614915	0.003377411	0.003661009	0.003364273	0.003599212	0.003036252
3	Proline	0.026426878	0.026928239	0.026586154	0.030505787	0.030603893	0.027533643
4	Valine	0.000908831	0.000497976	0.000755105	0.000730169	0.000620251	0.001143337
5	Betaine	0.426104093	0.424652311	0.438032736	0.421827797	0.398365213	0.423594509
6	Threonine	0.00046983	0.000624121	0.000469167	0.000461441	0.000661722	0.000486581
7	Homoserine	0.00398444	0.00462183	0.005017336	0.004814261	0.005469954	0.006158696
8	Pipecolic acid	0.009948074	0.008941329	0.011101124	0.010631345	0.010622063	0.010357807
9	Creatine	0.084543429	0.079717872	0.081844842	0.084329872	0.073739944	0.080740797
10	Leucine	0.001534548	0.00174587	0.001645844	0.002272588	0.001919176	0.003006048
11	Isoleucine	0.001830689	0.002100326	0.001793286	0.002064886	0.00225488	0.002055354
12	Methylcysteine	0.001668723	0.001092559	0.000746874	0.001685527	0.001106099	0.001814826
13	2-Methylnicotinamide	0.001023032	0.00104078	0.000491355	0.000988904	0.001314363	0.00127152
14	Spermidine	0.003031753	0.003933768	0.003929411	0.003993446	0.004679883	0.003376969
15	Glutamate	0.003764289	0.004284749	0.003890046	0.003964896	0.003717268	0.003900753
16	Guanine	0.000743826	0.00072316	0.001047843	0.000696623	0.001007719	0.000975372
17	2-Aminooctanoic acid	0.000944872	0.000826717	0.000623051	0.000796548	0.001192371	0.002315036
18	Phenylalanine	0.000927937	0.00144215	0.000869624	0.001125231	0.001271378	0.001274835
19	Citrulline	0.000647862	0.000354109	0.000449842	0.000604192	0.000410474	0.000579404
20	5-Methoxytryptophan	0.00251546	0.005049262	0.004333804	0.004289653	0.004570908	0.00402599
21	Deoxycytidine monophosphate	0.001938376	0.002088511	0.002298956	0.002435681	0.002570909	0.002010785
22	Guanosine monophosphate	0.002460748	0.002420032	0.002253865	0.002188366	0.002758588	0.003342713

23	Guanosine triphosphate	0.000652639	0.000818029	0.000572592	0.000553515	0.00093295	0.000863764
24	NADH	0.001211919	0.001280212	0.001192065	0.001147714	0.001246253	0.001128603
25	Dephospho-CoA	0.00117805	0.001369869	0.00097448	0.000883983	0.000769487	0.000718269
26	NADPH	0.001889309	0.001425817	0.001371715	0.001579891	0.001600423	0.001821456
27	Acetyl-CoA	0.039883482	0.031497942	0.036431154	0.031790542	0.0283699	0.027728865
28	Butyryl-CoA	0.001795082	0.001666291	0.00198582	0.001709081	0.001200847	0.001421067
29	3-Hydroxybutyryl-CoA	0.000546688	0.000327177	0.000421213	0.000277828	0.000305434	0.000312134
30	Betaine aldehyde	0.000826328	0.000360016	0.000828827	0.000462869	0.000399273	0.000632077
31	Homocysteine	0.000723852	0.000466006	0.000568655	0.000641664	0.00048494	0.001081455
32	Carnitine	0.006674024	0.008381844	0.006212621	0.007180351	0.007558647	0.007046403
33	Glucosamine	0.001013045	0.001025143	0.001117628	0.000961068	0.001038292	0.00094922
34	Acetylcarnitine	0.00519332	0.005612222	0.005214164	0.005074781	0.00479794	0.005337291
35	Glycerophosphocholine	0.044030322	0.049762857	0.041405545	0.046964922	0.052550305	0.0492843
36	S-Methyl-5-thioadenosine	0.059879501	0.063941101	0.062412508	0.057671211	0.068775514	0.057829859
37	Glutathione	0.008784353	0.009257559	0.008177327	0.009849786	0.008654454	0.008394538
38	Uridine monophosphate	0.000468528	0.000771116	0.000580823	0.000783343	0.00100227	0.001042042
39	Adenosine monophosphate	0.03904543	0.042882238	0.037683699	0.038614017	0.043892824	0.043317143
40	NAD^+	0.000793762	0.000449325	0.000572592	0.000570288	0.000484335	0.000606661
41	$NADP^+$	0.001071231	0.00128334	0.001309804	0.001127729	0.001322839	0.001176119
42	Hydroxyproline	0.001534548	0.00174587	0.001645844	0.002272588	0.001919176	0.003006048
